# Performance of Asbestos Enclosure Ventilation: Laboratory Evaluation of Complex Configuration

**DOI:** 10.1093/annweh/wxab041

**Published:** 2021-07-06

**Authors:** Ilpo Kulmala, Markku Linnainmaa, Anna Kokkonen, Kimmo Heinonen, Tomi Kanerva, Arto Säämänen

**Affiliations:** 1 VTT Technical Research Centre of Finland Ltd, FI-33101 Tampere, Finland; 2 Finnish Institute of Occupational Health, FI-33032 Työterveyslaitos, Finland; 3 Department of Environmental and Biological Sciences, University of Eastern Finland, FI-70211 Kuopio, Finland

**Keywords:** asbestos, asbestos enclosure, asbestos enclosure ventilation, ventilation efficiency, local air change index

## Abstract

The aim of the study was to find out good practices for effective air distribution inside a complex shaped asbestos enclosure and for control of pressure differences between the enclosure and the surroundings. In addition, sufficient pressure difference for asbestos containment was tested. The effect of air distribution was studied in laboratory conditions by constructing an L-shaped asbestos enclosure and connecting it to a negative pressure unit. The efficiency of six different ventilation configurations was compared using a tracer decay method and the local air change indexes as the performance indicator. The sufficient negative pressure for containment was assessed by simulating person traffic to and from the enclosure and recording the pressure difference continuously. The effect of a pressure controller unit in maintaining the target pressure difference was also tested by simulating filter loadings of the negative pressure unit causing changes in the air flow rate. The results showed that high nominal air change rates alone do not guarantee good air distribution. Effective air distribution within an asbestos enclosure can be arranged by locating additional air supply openings far away from the air exhaustion point, using recirculation air with a pressure controller, or extending the exhaust location to the poorly ventilated areas. A pressure difference of at least −10 Pa is recommended to ensure a sufficient margin of safety in practical situations.

**What’s Important About This Paper?**

During renovation work, asbestos-containing materials are still frequently encountered and can release asbestos fibres into the air when removed, causing inhalation hazards both for the workers and others in the vicinity of the work site. Our work addresses the performance of ventilated containment systems, and their effectiveness to reduce the risk of asbestos exposure. Ventilated containment systems can be used to control exposures during other activities involving hazardous materials, not just removal of asbestos-containing materials.

## Introduction

The use of asbestos has been banned or restricted in many countries. However, asbestos was widely used in buildings until mid-1980s, and during renovation work, asbestos-containing materials are frequently encountered. The removal of asbestos can release asbestos fibres into the air causing inhalation hazards both for the workers and for the occupants in the vicinity of the work site. To prevent the spread of asbestos fibres into the environment, the work is usually carried out by building a temporary enclosure around the work site and keeping it under negative pressure (SLIC, 2006). The workers inside the enclosure protect themselves by using effective respiratory protective equipment (PPE) and coveralls.

The passage to and from the working area in the enclosure takes place through a three-chamber airlock. Ventilation of the enclosure is provided by a negative pressure unit (NPU) equipped with high-efficiency particulate (HEPA) class filters, which effectively remove dangerous asbestos fibres from the exhaust air. By means of enclosure and negative pressure, the spread of asbestos fibres into the surrounding environment can be effectively prevented if all arrangements have been conducted correctly. In practice, however, problems have arisen in the renovation sites with relation to both the pressure difference and ventilation (Kokkonen *et al.*, 2019b). For example, excessively high negative pressure and ineffective dilution ventilation due to poor air distribution were encountered (Kokkonen *et al.*, 2019b). It is well established, that within the enclosure, the air pollutant concentrations depend on emissions, ventilation rate and air distribution.

Removal and dismantling of asbestos-containing material is highly regulated (Council Directive 2009/148/EC). In the Finnish guidelines for asbestos enclosure and negative pressure, the pressure difference between the enclosure and adjacent areas should be at least −5 Pa for asbestos materials other than crocidolite and at least −10 Pa for crocidolite (Ministry of Social Affairs and Health, Finland, 2015: Government Degree 798/2015/MSAH). A negative pressure requirement of at least 5 Pa is also set in the UK (HSE, 2012). However, a pressure difference of −5 Pa is relatively small, and is susceptible to, e.g., high wind speed or elevator movements (SLIC, 2006). In addition, door traffic is known to cause fluctuations in pressure differences (e.g. Hayden *et al.*, 1998; Tang *et al.*, 2005). Higher than 5 Pa negative pressure requirements have been set, e.g., in Belgium, France, Germany, and the Netherlands: At least 20 Pa is required in France (Danet *et al.*, 2000) and, similarly, a negative pressure of 20 Pa is stated to be generally sufficient in German guidelines (BAuA, 2014) and in the Netherlands (SZW, 2016). Belgium legislation says that the negative pressure should be between 10 and 40 Pa (SPF, 2017). According to BAuA (2014), a negative pressure of 50 Pa should not be exceeded.

There are no statutory guideline values for the air change rate in Finland, but according to the information provided by the Regional State Administrative Agency (AVI, 2015), the air in the enclosure must be replaced at least 10 times per hour during removal of asbestos materials other than crocidolite and at least 20 times per hour for crocidolite. In Germany, the minimum requirement for air change rate is eight changes per hour (BAuA, 2014). The same guideline is set for enclosures larger than 120 m^3^ in the UK (HSE, 2013), while ventilation must be at least 1000 m^3^ h^−1^ for enclosures <120 m^3^ (HSE, 2013). In the Netherlands, the air change rate is six times per hour (SZW, 2016). According to Danet *et al.* (2000), the minimum air change rate is four changes per hour in France and Belgium (SPF, 2017).

The arrangement of the ventilation is of great importance. There are general instructions for arranging the replacement air for the asbestos enclosure and exhaustion point location (e.g. Danet *et al.*, 2000; HSE, 2012), but only a few studies with experimentally verified ventilation performance (Pocock *et al.*, 2013). In practice, replacement air often comes only through the airlock. Field measurements have shown that this can in several occasions lead to poor ventilation. In addition, Pocock *et al.* (2013) showed that there are large differences in ventilation efficiency depending on the location of exhaustion within the enclosure. Poor mixing of air causes high concentrations and slow removal of contaminants within the enclosure (e.g. Burgess *et al.*, 2004), possibly increasing the risk of exposure.

Ventilation of enclosures requires high air change rates, which means large airflows. This in turn means that during the heating season, large amounts of warm indoor air are exhausted outdoors. To avoid the subsequent cooling of the building, a corresponding amount of cool replacement air must be heated. In building renovation sites, heating is usually done by unit heaters. This can lead to high energy consumption and, at the same time, difficulties especially in old buildings, where the electrical circuits may be overloaded due to the additional devices.

In residential buildings, asbestos removal often occurs in relatively small, confined spaces like in bathrooms or kitchens or in the basement boiler rooms. Our experience is that quite often the shape of the enclosure might be something else than a simple rectangular. Arranging enclosures and airlocks with well-performing ventilation for such complex situations may be challenging. The aim of these experiments was to study the effect of air distribution and exhaust on the efficiency of the ventilation in the complex shaped enclosure. In addition, this study aims to explore which pressure difference is sufficient for asbestos containment. The effect of pressure controller unit operation in maintaining the target pressure difference was also tested.

## Materials and methods

### Experimental setting

**Figure 1.**
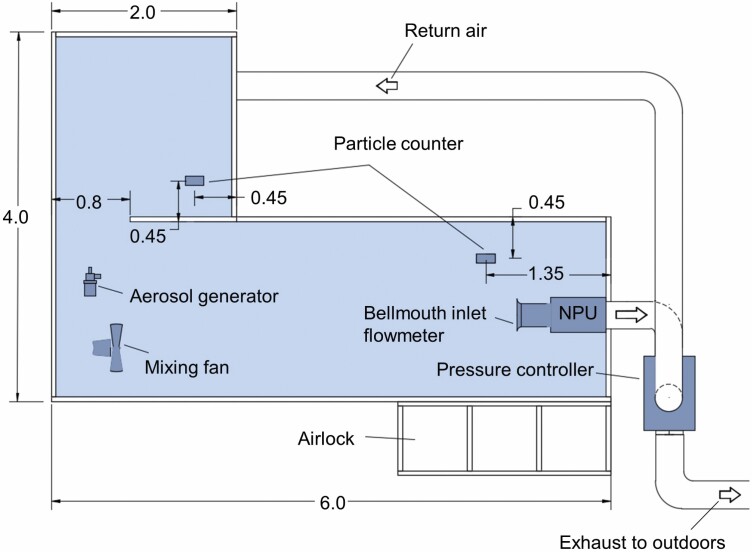
Floor plan of the enclosure and setup for the experiments. Dimensions in metres.

For the experiments, a complex L-shape enclosure with a floor area of 16 m^2^ and a volume of 32 m^3^ was constructed inside a large office room (Fig. 1). This kind of enclosure configuration is commonly found, e.g., in engineering and utility services rooms in both commercial buildings and private apartment houses. The enclosure was made of timber frames (48 × 48 mm) and 0.2-mm polyethene sheeting. The plastic sheets were stapled to the timbers and sealed with tape. The enclosure had a three-chamber airlock, each of which had the dimension of 0.8 m × 0.8 m × 2.0 m. The airlock doors were made of vertical slits in the plastic wall. Flaps to the doors were made of 0.8 m × 1.9 m plastic sheets, which were attached from above. To the bottom of the flaps was stapled a 50 mm × 50 mm timber batten weighing about 0.8 kg.

Ventilation of the enclosure was arranged with a NPU (Lifa HC 1100, Lifa Air Ltd, Finland). The device had an adjustable fan speed. The exhaust air was discharged outdoors. In some of the experiments, a pressure controller (Strong PT 315, Strong Finland) was attached to the NPU. The pressure controller is intended to be used in conjunction with a NPU to provide a constant, target pressure difference in the ventilated enclosure. The pressure controller automation ensures maintenance of the pressure level by adjusting the return air to exhaust air ratio. The NPU and controller were connected to a spiral wire-reinforced fabric hose with a diameter of 300 mm.

**Figure 2.**
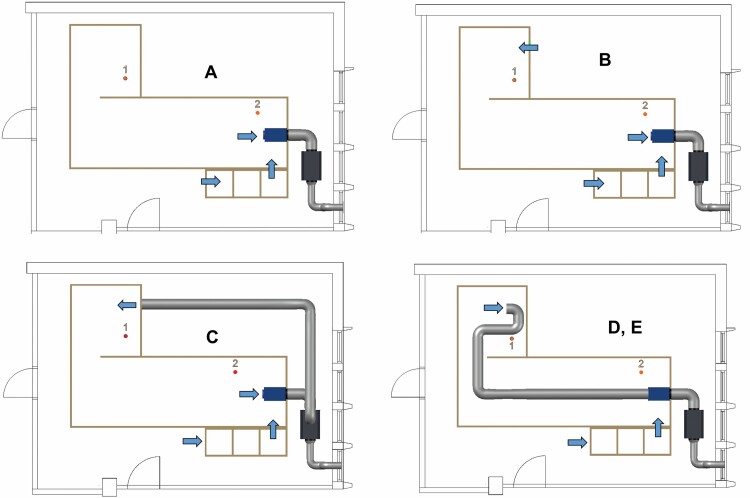
Investigated configurations.

The aim of the measurements was to find out how the ventilation performed and how air change efficiency in the enclosure could be improved. Therefore, the experimental setup attempted to create real-world conditions with a presumed good ventilation area (near the NPU, measuring point 2) and a location where air change was envisioned to be weaker (a separate 2 m × 2 m confined space connected to a larger space with an open 0.8 m × 2 m opening (measuring point 1). The studied configurations were (Fig. 2):

- replacement air through airlock, exhaust by NPU close to the airlock with a nominal air change rate (ACH) of 10 h^−1^; case A. Nominal ACH is defined as air flow rate of NPU (in m^3^ h^−1^) divided by the volume of the enclosure (in m^3^)
- replacement air through an opening in the enclosure wall (42% of the exhaust air) and airlock (58%), exhaust near airlock with ACH of 10 h^−1^; case B
- a pressure controller attached to the NPU; replacement air through the airlock (50%), and 50% through a 300-mm-diameter hose connected to the enclosure at the height of 1.35 m with nominal ACH of 10 h^−1^ (case C1) and with 19 h^−1^ (case C2, 75% recirculated air)
- replacement air through the airlock, exhaust from the enclosure with a 300-mm-diameter steel wire hose connected to the NPU with ACH of 10 h^−1^; exhaust at floor level (case D) and at the height of 1.35 m (case E).

### Measurements and data analysis

The actual airflow of the NPU was determined by measuring the pressure difference of a custom-built standardized Bellmouth (CEN ISO, 2017: ISO 5801) connected to the inlet of the device. The pressure difference was measured with a multi-function ventilation meter (TSI Velocicalc Plus 9555, TSI Instruments Ltd, UK).

The local ventilation efficiencies were measured using the tracer decay method, where submicron test particles were introduced into the enclosure and the local ventilation factor was determined from the decay rate of the particle concentration at two different points based on the submicron particle decay rate. Di-ethyl-hexyl-sebacat (DEHS) particles were generated by feeding air through a Laskin nozzle submerged in a bottle containing DEHS liquid. The measured count mean diameter of the produced aerosol was 0.5 µm with a standard deviation of 0.3 µm. The DEHS vapour pressure is very low (1 µPa at 273 K), so the test aerosol evaporation during the measurements was negligible in the studied particle size range. The time-dependent concentrations of the test particles within the enclosure were monitored using two direct-reading particle counters (Grimm models 1.108 and 11-C), connected to computers that stored the concentrations at 6-s intervals. These particle counters show good accuracy in different size ranges (Linnainmaa *et al.*, 2008).

In the air change efficiency measurements, tracer particle concentrations were increased to levels that were more than a few hundred times higher than the background concentration. Released particles were mixed well with an oscillating desk fan located in the corner of the enclosure near the tracer particle release point. Once the desired concentration was reached, the particle generator was turned off and the mixing fan was allowed to run for 1 min, after which the NPU was started. The decay in the particle concentration was measured over time using the particle counters. Measurements were continued for as long as *t*·λ > 1, where *t* is the length of the measurement period and λ is the measured air change rate, according to the ASTM (2007) standard guidelines. The measurement period was typically between 15 and 30 min. There were no people inside the enclosure during the measurements.

The size ranges of the particle counters were slightly different: the Grimm 1.108 measures particles in the size range of 0.30–20 µm in 15 channels, while Model 11-C measures particles in 31 channels in a size range of 0.265–34 µm. The performance of the ventilation was measured in the range 0.30–0.65 µm (model 1.108) and 0.265–0.675 µm (model 11-C). Particles in this size range follow air movements closely and behave similarly to gaseous tracers (Bivolarova *et al.*, 2017). To compare the particle counters with each other, they were placed in parallel to measure the same situation where the concentration was first raised and after which the NPU started, so that the concentrations fell back to the starting position. The readings of the meters differed somewhat so that Grimm 1.108 showed about 13% more than Grimm 11-C, but the linear dependence between them was good (correlation *R*^2^ > 0.99).

Assuming a uniform concentration and noting that there was no recirculating filtration, the time-dependent particle concentration C within the enclosure can be represented by the equation (Nazaroff, 2004):


$$\begin{array}{l} V\frac{dC}{dt}=G-qC-\;\beta VC \end{array}$$

where *V* is the volume of the enclosure, *G* the generation rate of the DEHS particles, *q* the exhaust air flow rate, and *β* the particle deposition rate onto indoor surfaces. During the decay phase, the particle generation *G* = 0, thus the equation (1) can be solved to give:


$$\begin{array}{l} ln\;C-ln\;C_{0}=-\left(\lambda +\beta \right)t \end{array}$$

where the air change rate *λ* is defined as *q*/*V* and *C*_0_ the concentration after the end of the particle feed at the time the NPU was started. By fitting the measured logarithmic concentrations with a linear graph, the least squares method can be used to solve both *C*_0_ and *λ* + *β*. Thus, the particle tracer decay method determines the combined effect of deposition and ventilation. In order to calculate the ventilation coefficient, the deposition rate must be taken into consideration. To determine this experimentally, the change in particle concentration was measured in the absence of ventilation after the end of the particle feed. In this way, the defined average deposition rate of about 0.27 h^−1^ was obtained. This value was then subtracted from the measured aerosol decay rates.

The experiments were carried out mainly with an exhaust flow rate of 0.090 m^3^ s^−1^, corresponding to a nominal ACH of 10 h^−1^. In addition, one set of measurements was made with the maximum airflow of 0.170 m^3^ s^−1^ (ACH 19 h^−1^). Each situation was measured three times.

To compare cases with different nominal air change rates, a local air change index, $\varepsilon_{p}^{a}$, is used. The local air change index utilizes the age of the air concept. It will represent the ability of a system to exchange the air in the enclosure (Mundt et al., 2004).


$$\begin{array}{l} \varepsilon_{p}^{a}=\frac{\tau_{n}}{{\bar{\tau }}_{p}}\times 100\%, \end{array}$$ (3)

where the nominal time constant *τ*_*n*_ = *V*/*q* = 1/*λ* and the local mean age of air, *τ*_*p*_, is approximated based on measured local air change rate


$$\begin{array}{l} {\bar{\tau }}_{p}=\frac{1}{\lambda_{p}}. \end{array}$$ (4)

The control of pressure differences was assessed in two cases: by entry and exit from the enclosure to the surrounding environment and filter loadings. Duration of entry or exit took 25 s and the waiting time to stabilize pressure inside the enclosure was 30 s. The person carried a toolbox during passages to represent a real-world situation. The test was carried out five times with initial pressure differences of both 10 and 5 Pa.

The effect of filter loadings on the pressure difference between the enclosure and surroundings was studied in two different situations: the NPU discharging directly outdoors and pressure controller attached to the NPU, so that part of the exhaust air was returned to the enclosure. The filter loading was simulated by adding a sheet of G4 class coarse filter media one at a time (one per minute), with total loading of 15 filter sheets. The initial enclosure pressure difference in these tests was 10 Pa. The pressure differences between the enclosure and surrounding environment were monitored at 1-s intervals using the multi-function ventilation meter (TSI Velocicalc Plus 9555, TSI Instruments Ltd, UK). In these experiments, the NPU air flow rate was 0.17 m^3^ s^−1^ and the supply air came through the airlock. The open area of the outermost airlock door opening was about 0.08 m^2^ when NPU was exhausting directly outdoors and about 0.04 m^2^ when the pressure controller was used.

### Statistical analysis

Comparison of investigated cases and measurement locations was performed using the GLM Univariate procedure with two-factor full factorial design (IBM SPSS Statistics 25, IBM Corp.). The ventilation performance indicator, the local air change index, was used in the statistical analysis. *Post hoc* pairwise comparison of means was done using the Tukey *B* test.

The effect of measurement location on the local air change index was studied by firstly calculating the difference of indexes in the two locations and then comparing the difference in different cases using one-way procedure and the Tukey *B* test in *post hoc* pairwise comparison of means.

## Results

The results of air change rates are summarized in Table 1. The mixing of air was poorest in the situation where the exhaust was near the airlock and replacement air came only through the airlock (case A). The local air change rate was lower than the nominal air change rate 10 h^−1^ at both measurement points. The situation was greatly improved by supplying about half of the replacement air through a 0.34 m × 0.34 m size G4-grade filter on the enclosure wall, so that at both measurement points, the local air change rate was greater than the nominal ACH (case B). Similar improvement was achieved by supplying the pressure controller’s return air as replacement air into the compartment (case C).

**Table 1.** Air change rate measurement results.

Case	Exhaust	Replacement air	ACH^a^ (h^−1^)	λ _p_ + sd^b^ (h^−1^) Measuring point	
				1	2
A	Near airlock	Airlock	10	5.6 ± 0.4	7.1 ± 0.6
B	Near airlock	Through G4 filter on the wall and airlock	10	11.2 ± 1.3	11.4 ± 0.7
C1	Near airlock	Through hose on the wall and airlock	10^c^	9.2 ± 0.5	10.4 ± 1.2
C2	Near airlock	Through hose on the wall and airlock	19^d^	19.0 ± 0.8	19.3 ± 0.9
D	Compartment, floor level	Airlock	10	10.4 ± 0.2	14.3 ± 0.3
E	Compartment height 1.35 m	Airlock	10	12.1 ± 0.5	14.0 ±0.6

**Figure 3.**
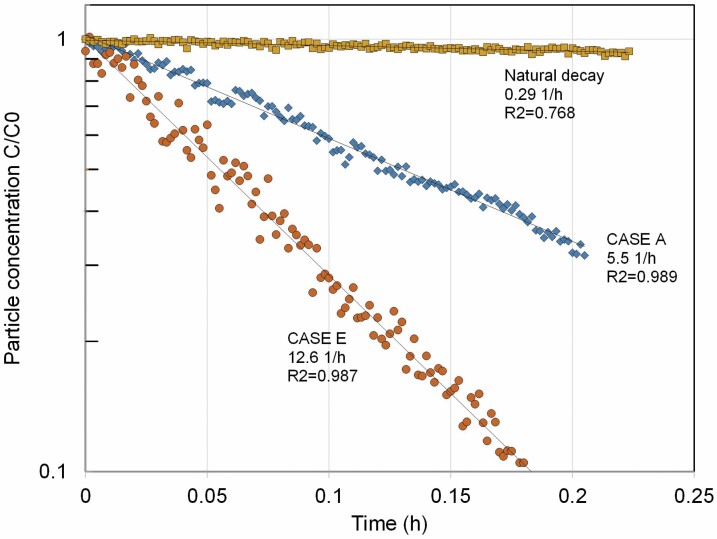
Example of measured concentration decays in the enclosure at measurement point 1 with two different ventilation arrangements and the same nominal air change rate. Case A: exhaust near airlock, Case E: exhaust in the small room with duct. The deposition rate of test aerosol without any ventilation is also shown.

Figure 3 shows an example of the measured concentrations. Only cases where the correlation coefficient *R*^2^ for the logarithmic decay curve fit was >0.95 were taken into account in the analysis.

^a^Nominal air change rate λ (NPU airflow rate divided by volume of the enclosure).

^b^λ _p_ is the local air change rate and sd = standard deviation.

^c^Partly HEPA-filtered air (50% recirculated air).

^d^Partly HEPA-filtered air (75% recirculated air).

The ventilation efficiency was also improved by moving the air extract point in the compartment using flexible ducting connected to the NPU (case D). In this way, the distance between the replacement air through the airlock and exhaust was maximized, resulting in high local air change rates both in the compartment and in the tunnel near the NPU. In the compartment, rising the exhaust level to 1.35 m further improved the situation (case E).

An example of the tracer particle concentration decays at the same air extraction rate but different exhaust air location is presented in Fig. 3, The results clearly show how the air distribution has a significant effect on the concentrations in the enclosure: with an optimal supply and exhaust combination the speed at which concentration decays could be doubled compared with the inefficient arrangement.

**Figure 4.**
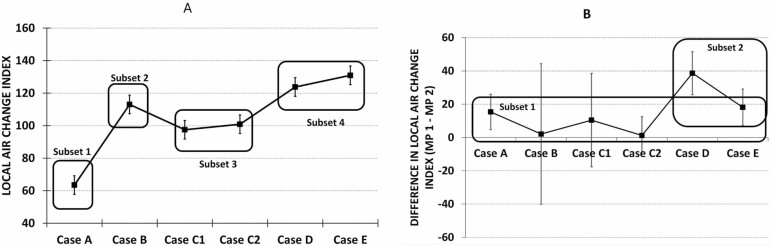
(**A**) The mean values and 95% CI of the local air change indexes in different cases and four homogeneous subsets based on Tukey *B post hoc* test (*P* = 0.05). (**B**) The difference in local air change indexes between measuring points MP 1 and MP 2 (mean and 95% CI) and two homogenous subsets based on Tukey *B* test.

The performance of the different air distribution cases within the enclosure was studied by comparing the local air change indexes. The analysis of variance showed that both the case and the location, as well as their interaction, had a significant (*P* < 0.01) influence on the local air change index. Four homogeneous subsets (*P* < 0.05) were formed (Fig. 4A). Case A formed its own subset 1 and had the lowest local air change index, the mean being 64%. The highest local air change indexes were obtained in cases D and E, which formed homogeneous subset 4. In this group, the mean of the local air change indexes was clearly over 100%. Two other subsets were formed from case B alone (subset 2) and cases C1 and C2 together (subset 3).

Similarity of the local air change index in different locations was examined with one-way analysis of variance and a Tukey *B post hoc* test for multiple comparisons of means (Fig. 4B). When the difference of the local air change indexes measured in different locations were compared, the cases D and E formed its own group (subset 2, Fig. 4B) compared with all others. The highest differences in the local air change indexes between locations were obtained from case D followed by case E. In the Tukey *B* test, they formed one homogenous group. However, case E did not differ significantly from the rest of the cases either. In other words, the case E belongs to both homogeneous groups (subsets 1 and 2).

**Figure 5.**
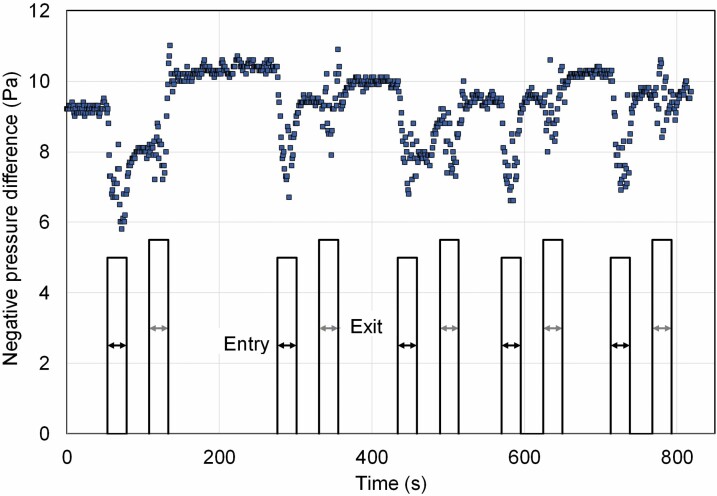
Variations in the pressure difference between the enclosure and surrounding environment during entry and exit.

Another key feature of the asbestos enclosure ventilation is its ability to maintain the pressure difference between the interior and the surroundings. Person movement to and from the enclosure decreased the negative pressure for a short time (Fig. 5). Negative pressure was adjusted in 10 Pa prior to the passage, while the lowest pressure difference was −5 Pa during person entry to the enclosure.

When the tests were repeated with an initial pressure difference of −5 Pa, the lowest pressure differences observed were between −1.4 and −2.9 Pa during ingresses and between −2.4 and −2.6 Pa during egresses.

**Figure 6.**
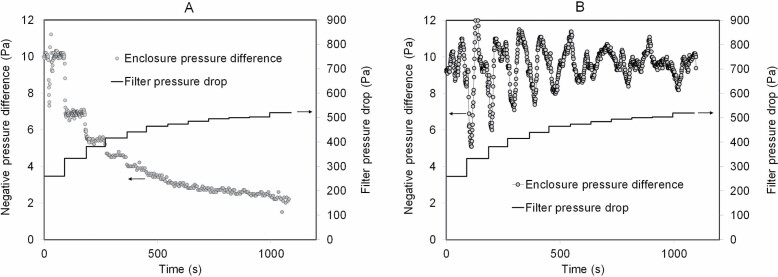
Effect of NPU’s filter loading on the negative pressure of an enclosure: (**A**) NPU and (**B**) NPU + pressure control unit.

Figure 6A and B represent the effect of NPU’s filter loading on the pressure differences between the enclosure and surrounding environment with (i) NPU discharging directly outdoors and (ii) pressure controller unit attached to NPU. When exhausting directly outdoors, the enclosure pressure difference decreased steadily with increasing filter loading (Fig. 6A). When a pressure controller was used, the enclosure pressure fluctuated somewhat, but the mean difference remained at the adjusted target level of −10 Pa during the test (Fig. 6B).

## Discussion

It was noticed that both the air flow rate and the air distribution within the asbestos enclosure were of crucial importance in the performance of the ventilation. The ability of the ventilation to dilute the contaminants inside the enclosure depends on the local air change rate. Naturally, the increased air flow rate (case C2 compared with case C1) made the dilution more effective. The arrangement of the distribution of the replacement air also plays an important role in the effectiveness of dilution. Based on the local air change index, air supply through an airlock and exhausted close to the airlock turned out to be the worst method so that the dilution inside the enclosure was much worse (ε ^*a*^_*p*_ = 64%) than in the perfect mixing case (ε ^*a*^_*p*_ = 100%). The cases where the distance between the inlet of the replacement air and the exhaust was longest (cases D and E) showed the best effectiveness. In these cases, the local air change indexes were >100%, indicating a displacement or piston flow type air flow pattern. When the replacement air was supplied to the enclosure via two inlets (cases B, C1, and C2), the local air change indexes were close to 100% indicating perfect mixing.

The results are similar to those observed by Pocock *et al.* (2013) who investigated the factors that affect the airflow characteristics of ventilated asbestos enclosures including ventilation rate, location of extract position, and number of airlock inlets. They presented tracer gas measurement results for two different enclosures with volumes of 60 and 87 m^3^ and found the importance of the position of the NPU to achieve good mixing of air within the enclosure.

Efficient ventilation is essential to control airborne asbestos within enclosures. In good ventilation, the working areas where fibres are released are flushed well with air, diluting concentrations effectively. The results show, however, that high nominal air change rates alone may not guarantee good air distribution. Inefficient ventilation leads to increased fibre levels in the enclosure and thus dust deposits on surfaces.

In actual work place situations, air exchange in poorly ventilated areas could be improved by drawing make-up air from outside the enclosure through disposable polythene air ducts to targeted locations. The air flow in the plastic duct is then driven by the pressure difference between the enclosure and surroundings. However, a damper or other device needs to be incorporated in such a supply air system to prevent reverse flow as a result of fan failure or adverse wind conditions (HSE 2012).

Asbestos sites have widely varying geometries and sizes; however, the studied configuration is similar to that commonly found in asbestos work in apartments. Additional air inlets in the fixed walls are not always possible. The results showed that good alternatives are to distribute air through hoses to poorly ventilated zones or to move the extract point using a hose to these locations, as recommended by current guidelines (Danet *et al.*, 2000; HSE, 2012). This is particularly important if the airlock and extract unit are to be sited close to one another, which may be the case in complex real-world settings.

With large enclosures, the airflow rates also need to be large. In these cases, the airlock alone may not supply sufficient replacement air and additional replacement air arrangements are required (Gibson, 2014). Moreover, during the building heating period, the ventilation energy consumption could be quite high. Exhausting the air outdoors 24/7 results in high energy consumption. A sustainable and at the same time safe alternative could be returning part of the HEPA-filtered exhaust air back to the enclosure. In this way, the ventilation efficiency can be improved while saving heating energy.

Another key parameter in asbestos enclosure ventilation is its pressure difference relative to the surroundings. It is not constant but varies due to environmental factors, entry to and exit from the enclosure, and changes in the NPU airflow because of filter clogging. In various guidelines, the recommended negative pressure difference is between 5 and 40 Pa (Danet *et al.*, 2000; HSE 2012; BAuA, 2014; Ministry of Social Affairs and Health, Finland, 2015: Government Degree 798/2015/MSAH; SZW, 2016; SPF, 2017). The tests with an initial pressure difference of −5 Pa showed that the effect of workers’ movements to and from the enclosure together with other possible interfering factors, such as high wind speed and direction or elevator movements, might decrease the pressure difference quite close to the zero level (SLIC 2006, Papadopoulos *et al.* 2018). A pressure difference of at least −10 Pa was instead found to meet the minimum requirement for a negative pressure of 5 Pa (Ministry of Social Affairs and Health, Finland, 2015: Government Degree 798/2015/MSAH) also during passages or other external factors, such as strong wind. Based on the results of this study, a pressure difference of −5 Pa may not provide a sufficient margin of safety and therefore at least −10 Pa is recommended.

The pressure controller proved to be efficient in maintaining the targeted negative pressure difference between the enclosure and the surroundings in case of filter loadings. Furthermore, it facilitates efficient air flow patterns inside the enclosure since the return air can be supplied to poorly ventilated locations.

Enclosures with HEPA filtration are also used in repair and removal work of other hazardous materials such as mould, creosote, lead, or other hazardous substances. These removals, however, are not as strictly controlled as the removal of asbestos-containing materials (Council Directive 2009/148/EC). Failures in enclosure containment during removal of hazardous substances other than asbestos have recently been reported (Kokkonen *et al*., 2017, 2019a). Common failures encountered included problems in achieving continuous negative pressure, no requirement set for negative pressure, and no monitoring of pressure differences between enclosures and surroundings (Kokkonen *et al*., 2017). To prevent exposure of construction workers and building users to harmful particles, asbestos enclosure requirements should be applied also for dismantling work containing hazardous substances other than asbestos.

## Conclusions

Ventilation in some parts of an asbestos enclosure may be poor, especially if there are compartments, which are far away from the air exhaust or supply replacement air. The situation could be greatly improved with properly located additional air supply openings, use of recirculation air with a pressure controller, or extending the exhaust location to the poorly ventilated areas. A pressure difference of at least −10 Pa is recommended to ensure a sufficient margin of safety. In addition to measuring pressure differences, air flow rates of NPUs should be measured in real-world conditions. The results are directly applicable also to other renovation or refurbishment work, which may release dangerous or harmful substances into the air.

## Data Availability

The data underlying this article will be shared on reasonable request to the corresponding author.
